# Multiscale Biology of Cardiovascular Risk in Psoriasis: Protocol for a Case-Control Study

**DOI:** 10.2196/28669

**Published:** 2021-09-28

**Authors:** Hannah Kaiser, Amanda Kvist-Hansen, Christine Becker, Xing Wang, Benjamin D McCauley, Martin Krakauer, Peter Michael Gørtz, Kristoffer Mads Aaris Henningsen, Claus Zachariae, Lone Skov, Peter Riis Hansen

**Affiliations:** 1 Department of Dermatology and Allergy Copenhagen University Hospital Herlev and Gentofte Copenhagen Denmark; 2 Department of Cardiology Copenhagen University Hospital Herlev and Gentofte Copenhagen Denmark; 3 Division of Clinical Immunology Department of Medicine Icahn School of Medicine at Mount Sinai New York, NY United States; 4 Department of Genetics and Genomic Sciences Icahn School of Medicine at Mount Sinai New York, NY United States; 5 Department of Clinical Physiology and Nuclear Medicine Copenhagen University Hospital Bispebjerg and Frederiksberg Copenhagen Denmark; 6 Department of Clinical Physiology and Nuclear Medicine Copenhagen University Hospital Herlev and Gentofte Copenhagen Denmark; 7 Department of Clinical Medicine University of Copenhagen Copenhagen Denmark

**Keywords:** cardiovascular disease, psoriasis, study protocol, cardiovascular imaging, proteomics, lipidomics, microbiome, mass cytometry, bioinformatics, system biology

## Abstract

**Background:**

Patients with psoriasis have increased risk of cardiovascular disease (CVD) independent of traditional risk factors. The molecular mechanisms underlying the psoriasis-CVD connection are not fully understood. Advances in high-throughput molecular profiling technologies and computational analysis techniques offer new opportunities to improve the understanding of disease connections.

**Objective:**

We aim to characterize the complexity of cardiovascular risk in patients with psoriasis by integrating deep phenotypic data with systems biology techniques to perform comprehensive multiomic analyses and construct network models of the two interacting diseases.

**Methods:**

The study aims to include 120 adult patients with psoriasis (60 with prior atherosclerotic CVD and 60 without CVD). Half of the patients are already receiving systemic antipsoriatic treatment. All patients complete a questionnaire, and a medical interview is conducted to collect medical history and information on, for example, socioeconomics, mental health, diet, and physical exercise. Participants are examined clinically with assessment of the Psoriasis Area and Severity Index and undergo imaging by transthoracic echocardiography, ^18^F-fluorodeoxyglucose positron emission tomography/computed tomography (^18^F-FDG-PET/CT), and carotid artery ultrasonography. Skin swabs are collected for analysis of microbiome metagenomics; skin biopsies and blood samples are collected for transcriptomic profiling by RNA sequencing; skin biopsies are collected for immunohistochemistry; plasma samples are collected for analyses of proteomics, lipidomics, and metabolomics; blood samples are collected for high-dimensional mass cytometry; and feces samples are collected for gut microbiome metagenomics. Bioinformatics and systems biology techniques are utilized to analyze the multiomic data and to integrate data into a network model of CVD in patients with psoriasis.

**Results:**

Recruitment was completed in September 2020. Preliminary results of ^18^F-FDG-PET/CT data have recently been published, where vascular inflammation was reduced in the ascending aorta (*P*=.046) and aortic arch (*P*=.04) in patients treated with statins and was positively associated with inflammation in the visceral adipose tissue (*P*<.001), subcutaneous adipose tissue (*P*=.007), pericardial adipose tissue (*P*<.001), spleen (*P=*.001), and bone marrow (*P*<.001).

**Conclusions:**

This systems biology approach with integration of multiomics and clinical data in patients with psoriasis with or without CVD is likely to provide novel insights into the biological mechanisms underlying these diseases and their interplay that can impact future treatment.

**International Registered Report Identifier (IRRID):**

DERR1-10.2196/28669

## Introduction

### Background

Psoriasis is a chronic inflammatory disease affecting 2% to 3% of the adult population and is associated with an increased risk of developing other inflammatory diseases, such as cardiovascular disease (CVD), inflammatory bowel disease, and diabetes [1,2]. Moderate-to-severe psoriasis is associated with increased risks of myocardial infarction and stroke independent of traditional risk factors, such as smoking, BMI, diabetes, hypertension, and dyslipidemia [3,4]. In addition, patients with psoriasis display increased prevalence of subclinical CVD, for example, endothelial dysfunction and increased carotid artery intimamedia thickness (CIMT), compared to controls [5-7]. Although the molecular mechanisms underlying the link between psoriasis and CVD remain to be identified in detail, they may rely, in part, on shared inflammatory pathways [8]. Indeed, psoriasis and atherosclerosis are both immune-driven chronic inflammatory diseases with an overlap of inflammatory mediators, including T-helper 1 (Th1) and Th17 cells [9]. It remains unclear, however, if treatment of psoriasis with systemic antipsoriatic medications can decrease the risk of CVD, but studies have indicated that tumor necrosis factor inhibitors and methotrexate may carry this potential [10,11].

**Figure 1:**
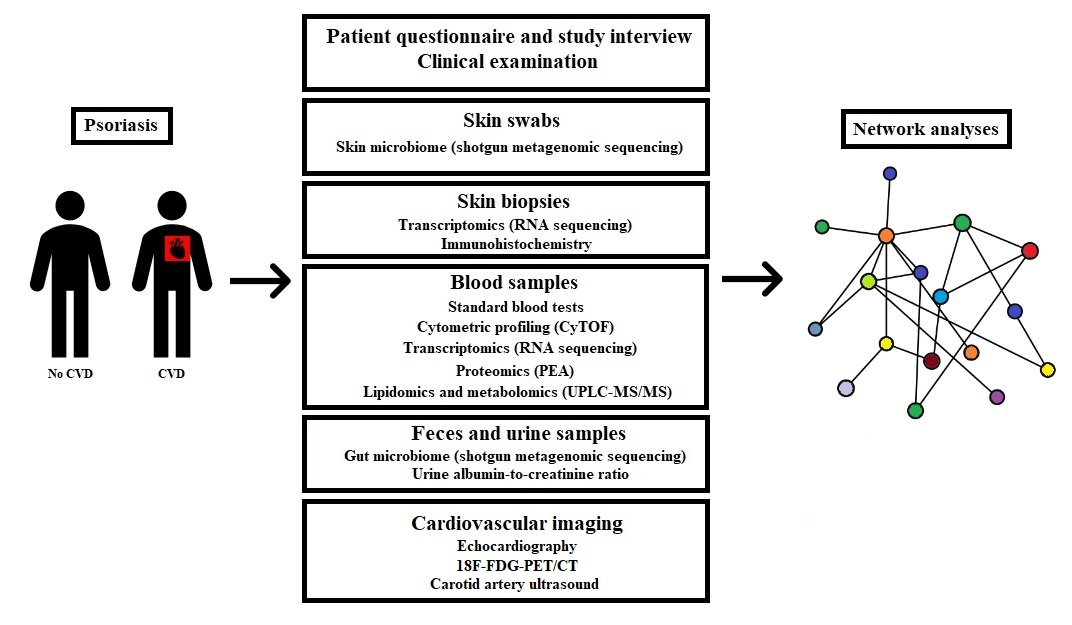
Overview of study examinations and analyses. ^18^F-FDG-PET/CT: ^18^F-fluorodeoxyglucose positron emission tomography/computed tomography; CVD: cardiovascular disease; CyTOF: cytometry by time-of-flight; PEA: proximity extension assay; UPLC-MS/MS: ultra-high performance liquid chromatography/tandem mass spectrometry.

Basic, translational, and clinical research directed separately at psoriasis or CVD has led to the identification of the molecular disease mechanisms and the development of new therapies aimed at psoriasis or CVD. However, this traditional paradigm is built on studies that do not capture deep phenotypes of individual patients, and to identify the central drivers of diseases and enable precision therapy, systems biology–oriented approaches are needed that seek to integrate all relevant available data. Advances in high-throughput molecular profiling and computational analysis techniques offer such novel opportunities to improve the understanding of disease connections and accelerate the discovery of new therapeutic strategies [12-15]. A multiscale biology approach has not, to our knowledge, been applied previously to provide comprehensive insights into the association between psoriasis and CVD. Therefore, we will use these techniques to investigate the pathophysiological links between psoriasis and CVD by integrating deep phenotypic data with multiomic data and use systems biology techniques to interrogate the molecular mechanisms connecting the two diseases (Figure 1). This approach will provide information on the fundamental regulatory circuits that drive psoriasis and CVD, enable the discovery and testing of novel biomarkers and therapeutics, and deliver other insights relevant for future prevention and therapy in these patients.

### Aims and Objectives

This study aims to investigate the complex multiscale interactions that drive cardiovascular risks in individual patients with psoriasis, involving specific gene transcripts, protein and lipid markers, signaling pathways, immune cell types, organ systems, and microbiota. Moreover, the study will examine multiscale differences across patient groups with psoriasis having distinct phenotypic traits, for example, with or without CVD, systemic antipsoriatic treatment or psoriatic arthritis, and early or late onset of psoriasis.

## Methods

### Study Population

The study will include 120 patients (aged ≥30 years) with moderate-to-severe plaque psoriasis, with 60 having prior (over 6 months before inclusion) atherosclerotic CVD, including myocardial infarction, coronary revascularization, ischemic stroke, and/or peripheral artery disease, and 60 not having this history. Furthermore, half of the patients receive systemic antipsoriatic treatment (unchanged therapy in the preceding 3 months), while the other half of the patients do not receive systemic antipsoriatic treatment. The exclusion criteria are shown in Textbox 1.

**Study exclusion criteria.**

**Exclusion criteria**

- Dementia or other major psychological or physical incapacities
- Other chronic systemic diseases
- History of cancer with throat or thoracic irradiation or history of cancer with <3 years recurrence-free control, and for hematologic cancers, <5 years recurrence-free control
- Major surgery, pregnancy, labor, or breastfeeding ≤6 months before inclusion
- Immobility
- Dysregulated diabetes (glycated hemoglobin >10%)
- Systemic treatment with prednisolone or antibiotics <1 month before inclusion
- Severe claustrophobia
- Severe kidney disease (glomerular filtration rate <30 mL/min)
- Inability to understand the information relating to participation in the study

### Patient Recruitment

Patients are recruited at the Department of Dermatology and Allergy, Herlev and Gentofte Hospital, when attending regularly scheduled visits for psoriasis control. Recruitment began in January 2018 and is also at the Department of Dermatology, Bispebjerg Hospital, at selected private dermatology clinics in the Copenhagen area, and through public advertisement of the research project on the home page of Herlev and Gentofte Hospital, social media outlets, and the member magazine of the Danish Psoriasis Association. All patient-related examinations are performed at Herlev and Gentofte Hospital. Because of the extensive examination program, the study visits are extended over 2 days of attendance within a period of 2 weeks.

### Patient Questionnaire and Study Interview

Each participant completes a questionnaire containing questions regarding civil status, occupation, educational level, annual household income, psychological stress, depression, self-rated health, diet, and exercise. The questionnaire also contains questions to establish the Dermatology Quality of Life Index. An interview is conducted by study physicians with questions regarding history of psoriasis, current and previous medical treatment of psoriasis, history of CVD, medical treatment of CVD, family history of CVD, comorbidities (diabetes, hypertension, hypercholesterolemia, etc), odontological status, smoking status, alcohol consumption, other medications including use of antibiotics (exclusion if taken within 1 month before consideration for inclusion), and ethnicity.

### Clinical Examination

A full skin examination is performed in each patient, and the severity of psoriasis is measured according to the Psoriasis Area and Severity Index and body surface area [16]. In addition, fingernails are examined for signs of psoriasis. Blood pressure is measured on each arm after the patient has rested for 5 minutes in the sitting position and is registered as the mean of these two measurements. BMI and waist-to-hip ratio are registered. The tongue is examined for the presence of a geographical tongue [17].

### Skin Swabs: Skin Microbiome

Isohelix DNA/RNA buccal swabs (SK-1S, Cell Projects Ltd) are used to collect samples from the skin. One swab is taken from a psoriasis plaque (if applicable), where the patient has at least 4 to 6 cm^2^ of affected skin. A second swab is taken from adjacent clinically healthy skin. The sample is taken by rubbing the skin with the swab for 60 seconds and is stored in DNA/RNA shield (Zymo Research Corp). Site-specific skin microbiomes vary between different body regions [18], and therefore, we attempt to collect samples from the same body site among patients in the following priority: the lumbar area and buttocks, arms, and legs. Sampling controls are collected by holding the swabs freely in the examination room for 3 minutes. The skin microbiome is analyzed by shotgun metagenomics with untargeted sequencing of all microbial genomes [19].

### Skin Biopsies: Transcriptomics and Immunohistochemistry

Two 4-mm skin punch biopsies are taken under local anesthesia, including one from active psoriatic skin and one from clinically healthy skin neighboring the psoriatic biopsy area with a minimum distance of 2 cm from the psoriasis biopsy. The biopsy locations have the same priority for body sites as the skin swabs. After collection, biopsies are immediately cut in two parts and processed separately for RNA sequencing and immunohistochemistry.

Biopsies for RNA sequencing are placed directly into RNAprotect Tubes (QIAGEN), which are stored at 4°C overnight and thereafter at −80°C. RNA is isolated from biopsies, and its quality is assessed using the 2100 Bioanalyzer (Agilent). Sequencing libraries are prepared using SureSelect XT RNA Direct (Illumina) for samples with an RNA integrity number (RIN) score >8 and are sequenced at the Genomics Core Facility at Icahn School of Medicine at Mount Sinai.

Biopsies for immunohistochemistry are placed into a cryomold (Tissue Tek, Sakura Finetek) with optimal cutting temperature (OCT) solution (Tissue Tek, Sakura Finetek) that is subsequently snap-frozen with liquid nitrogen and immediately stored at −80^°^C. OCT samples are cut, mounted, fixed, and stained with hematoxylin and eosin, and with antibodies to selected target antigens using standard immunohistochemical techniques.

### Blood Samples: Mass Cytometry, Proteomics, Lipidomics, and Metabolomics

Routine hematological and biochemical parameters, including high-sensitive C-reactive protein and N-terminal pro-brain natriuretic peptide, are analyzed at the Department of Clinical Biochemistry, Herlev and Gentofte Hospital, Denmark.

For mass cytometry profiling, blood is drawn in an ACD-A (acid citrate dextrose, Hettich Lab) tube, and 1 mL of blood is aliquoted into a tube containing 2 µL of Cell Activation Cocktail (phorbol 12-myristate-13-acetate [40.5 µM], ionomycin [669.3 µM], and Brefeldin A [2.5 mg/mL]; Biolegend). A second tube containing 1 mL of blood without the Cell Activation Cocktail is also collected. The tubes are mixed gently and then placed in an incubator at 37°C (5% CO_2_)_._ After 6 hours, 1.4 mL of proteomic stabilizer (PROT1, Smart Tube Inc) is added to each tube and mixed. The tubes are incubated for 10 minutes at room temperature and are then immediately placed at −80°C. Immune cell populations and cytokines are analyzed via mass cytometry by time-of-flight (CyTOF) [20] using a panel of 42 antibodies to profile various immune cells, including dendritic cells and T-cell subtypes, such as Th1, Th2, Th9, Th17, Th22, and Treg cells, in addition to relevant cytokines, such as interleukin (IL)-17 and IL-23.

For profiling of plasma proteins, blood is collected in ethylenediaminetetraacetic acid (EDTA) tubes and centrifuged for 10 minutes at 2000 rpm. The plasma is aliquoted into 1-mL tubes and immediately stored at −80^°^C. Plasma concentrations of proteins are measured using the Olink Proseek multiplex assay (Olink Bioscience), which uses proximity extension assay technology to detect protein biomarkers in liquid samples [21]. In brief, pairs of antibodies linked to oligonucleotides bind to their target protein in close proximity so that the oligonucleotides can hybridize and generate a unique sequence that can be detected and quantified by subsequent quantitative real-time polymerase chain reaction [22,23]. Predesigned Olink multiplex biomarker panels (Inflammation, Cardiovascular II, and Cardiovascular III) are used to determine protein profiles [24].

Lipidomic and metabolomic profiling of plasma are assessed by Metabolon (Morrisville), where approximately 1100 lipids and 5200 metabolites are measured by ultra-high performance liquid chromatography/tandem mass spectrometry [25].

For transcriptomic profiling, blood (2.5 mL) is drawn into PAXgene RNA Tubes (BD Bioscience), placed at −20°C overnight, and then stored at −80°C. RNA is isolated using the PAXgene Blood RNA Kit (QIAGEN), and its quality is assessed using the 2100 Bioanalyzer (Agilent). Sequencing libraries are prepared using TruSeq Stranded Total RNA kits (Illumina) for samples with a RIN score >8 and are sequenced at the Genomics Core Facility at Icahn School of Medicine at Mount Sinai. Owing to the relatively small number of study subjects, genomics are currently not planned to be assessed in this work.

### Feces Sample: Gut Microbiome

All patients receive an OMNIgene GUT kit (DNA Genotek) for collection of feces at a maximum of 14 days after the blood samples. The gut microbiome is analyzed by shotgun metagenomics [19].

### Urine Sample: Albumin-to-Creatinine Ratio

A urine sample is collected, and the albumin-to-creatinine ratio is determined at the Department of Clinical Biochemistry, Herlev and Gentofte Hospital, Denmark.

### Echocardiography

Comprehensive two-dimensional resting transthoracic echocardiography, including tissue Doppler imaging, is performed using a Vivid E-95 ultrasound machine (GE Healthcare) with a M5Sc-D (1.4-4.6 MHz) transducer to determine myocardial structural and functional indices, such as left ventricular mass and systolic and diastolic function, right ventricular function, and left ventricular global longitudinal strain. Epicardial and pericardial adipose tissues are also measured in standard parasternal and short axis views [26,27]. All analyses are performed and stored in EchoPAC version 203.82 (GE Healthcare).

### ^18^F-Fluorodeoxyglucose Positron Emission Tomography/Computed Tomography

At a maximum of 14 days after the blood samples are taken, all patients undergo ^18^F-fluorodeoxyglucose (FDG) positron emission tomography/computed tomography (PET/CT). In brief, subjects are injected with 3.5 MBq (0.09 mCi) per kilogram FDG after fasting overnight. A whole-body FDG-PET/CT is performed 120 minutes after FDG injection on a GE Discovery 710 scanner (GE Medical Systems) using the proprietary Q.Clear PET reconstruction algorithm. Anatomic localization and attenuation correction are provided by an unenhanced low-dose CT scan. Regions of interest (ROIs) are delineated around the aorta in consecutive 3-mm-thick axial PET and CT slices using MIM 6.9.2 software (MIM Software Inc). FDG uptake in aortic segments is quantified by calculating the mean of the maximum activity in each ROI normalized to the mean activity in the superior vena cava (maximum target-to-background ratio [TBR_max_]) to determine vascular inflammation according to established methodology [28]. Adipose tissue in brown, pericardial, visceral, and subcutaneous fat is measured by manually locating volumes of interest in the lateral neck, anterior to the pericardium at the level of the aortic root, caudal to the kidneys, and in the loin, respectively. Moreover, FDG uptakes in the bone marrow (lumbar vertebra L1-L5) and spleen are measured to assess activation of hematopoietic tissues. Separate electrocardiogram-gated low-dose CT acquisition is performed for determination of the coronary calcium score using a combined Agatston score for all coronary arteries using Siemens SyngoVia software VB40 (Siemens Healthcare) [29,30].

### Carotid Artery Ultrasound Imaging

Ultrasound imaging of CIMT is performed in the right and left common carotid arteries by using an Affiniti 70G ultrasound system with a 5-12 MHz linear array transducer (Philips Ultrasound Inc) with Philips Q-App IMT software (version 3.03). CIMT is measured according to the Mannheim consensus on a 10-mm far wall segment of the distal common carotid artery during diastole, avoiding areas with focal thickening [31]. The presence of atherosclerotic plaques is also assessed in both carotid arteries.

### Bioinformatics Analyses

For transcriptomics, RNA sequencing (RNA-seq) FASTQ sequence files are first subject to quality trim followed by alignment to the HG38 human genome and count summarization. Samples are normalized, and differential expression analysis is carried out to detect genes that are expressed at significantly different levels between groups. Gene set enrichment analysis is performed by the CAMERA method using reference data sets that include Hallmark, KEGG, Reactome, and Gene Ontology [32]. Protein-protein interaction network analysis is carried out using Cytoscape with the StringAPP plugin [33-35]. Functional enrichment analysis of the interaction network is performed using STRING enrichment against a collection of gene set databases [34,35].

For metagenomics, FASTQ files are quality trimmed before human genome mapping to filter out human reads. Microbial taxonomy classification is performed by the k-mer–based Kraken2 tool supplied with a microbial database including archaea, bacteria, fungi, protozoa, viruses, and vectors [36]. Relative taxonomy abundance is analyzed by LEfSe, and absolute abundance is analyzed by DESeq2 [37,38]. Alpha diversity is calculated using the phyloseq package in RStudio (R version 3.6) [39].

For high dimensional CyTOF, data are first preprocessed in Cytobank, and then, FCS data files are imported into RStudio [40]. Data are arcsinh-transformed and then scaled for heatmap presentation. Unscaled transformed data are used for FlowSOM hierarchical clustering [41]. The clusters are then manually annotated and visualized after UMAP dimension reduction [42]. Differential cell lineage markers are further quantified and cytokine levels are stratified by clustered cell populations.

For proteomics, plasma protein levels are measured using the Cardiovascular II, Cardiovascular III, and Inflammation panels (Olink Bioscience), and reported as normalized protein expression levels (NPX) in log2 scale. Proteins are filtered out when 40% of samples are below the limit of detection. Protein changes are analyzed with the *limma*-trend method for comparisons of interest. Functional enrichment analysis is performed as described above for transcriptomic data [43].

The strength of a systems biology approach is the potential to integrate data from multiple platforms. There are several examples of how this approach has been successfully applied in the disease areas of psoriasis and in particular CVD [44,45]. We will use previously described computational methods to integrate multiomics data sets to develop a comprehensive and integrated view of cardiovascular risk in patients with psoriasis [46-48].

## Results

The preliminary results of ^18^F-FDG-PET/CT data have recently been published, where vascular inflammation in patients treated with statins was significantly reduced in the ascending aorta (*P*=.046) and aortic arch (*P*=.04) compared to the findings in patients not treated with statins, even though most statin-treated patients were, of course, in the CVD group [49]. Moreover, we very recently reported positive associations between vascular inflammation and inflammation in the visceral adipose tissue (*P*<.001), subcutaneous adipose tissue (*P*=.007), pericardial adipose tissue (*P*<.001), spleen (*P=*.001), and bone marrow (*P*<.001) in our study population [50].

The study has been approved by the ethics committee of the Capital Region, Denmark (H-17003458) and the local data protection agency (ID: HGH-2017-103, I-suite: 05977). All participants will be asked to sign an informed consent form before entering the study. The study has been conducted in agreement with the Declaration of Helsinki.

The ethics approval for the study was granted in March 2017. Recruitment for the study began in January 2018 and was completed in September 2020. Analyses of transcriptomics and proteomics have been completed, while analyses involving metagenomics, lipidomics, metabolomics, mass cytometry experiments, and immunohistochemistry are underway. Data processing and statistical analyses began in September 2020, and the first results of the study were published in the beginning of 2021 [49,50].

## Discussion

This is an observational study with a case-control design that includes adult patients with psoriasis with or without atherosclerotic cardiovascular disease, and with or without systemic antipsoriatic treatment. The major strength of this study is the extensive number of examinations and samples collected from each patient to achieve deep phenotypic characterization. High-throughput molecular profiling technologies and computational analyses are utilized, and data are integrated by multiscale network analyses. With this approach, results are likely to shed light on new drivers and mechanisms of cardiovascular risk in psoriasis that can impact precision medicine [44,51]. Patients are specifically included with and without prior CVD, and with strict inclusion and exclusion criteria. Moreover, the psoriasis diagnosis is verified by dermatologists.

An important limitation of the study is that a control group without psoriasis is not included, which makes it impossible to compare the results with healthy individuals. Moreover, the computational analyses are data-driven and hypothesis-free, which precludes a priori sample size calculations. Another potential limitation is that patients with CVD are often older than those without CVD, so matching patients with and without CVD can be difficult. Furthermore, due to the extensive number of examinations and the 120 minutes of rest required for patients before the ^18^F-FDG-PET/CT scan, the scan is not performed on the day of clinical examination and collection of blood and skin samples, potentially leading to temporal changes in the state of systemic inflammation. All skin biopsies and swabs might not be taken from the same body regions in all patients due to variations in the location of psoriasis-affected skin. Moreover, because of the recruitment setup and study hospital localization, most patients will probably be recruited from the Copenhagen area, although patients from other parts of Denmark are eligible and can contact the project through social media or the member magazine of the Danish Psoriasis Association.

## Abbreviations

CIMT: carotid artery intimamedia thickness
CVD: cardiovascular disease
CyTOF: mass cytometry by time-of-flight
FDG: fluorodeoxyglucose
IL: interleukin
OCT: optimal cutting temperature
PET/CT: positron emission tomography/computed tomography
RIN: RNA integrity number
ROI: region of interest
Th: T-helper
